# Can Dawul Kurundu (*Neolitsea involucrate*) leaf extract be used as a plant‐based stabilizer in set yoghurt production?

**DOI:** 10.1002/fsn3.3859

**Published:** 2023-11-22

**Authors:** Wimukthika Wijekoon, Udayagee Kumarasinghe, Amali Alahakoon, Shishanthi Jayarathna, Hasitha Priyashantha

**Affiliations:** ^1^ Faculty of Graduate Studies University of Sri Jayewardenepura Nugegoda Sri Lanka; ^2^ Department of Biosystems Technology, Faculty of Technology University of Sri Jayewardenepura Pitipana Homagama Sri Lanka; ^3^ Department of Molecular Sciences Swedish University of Agricultural Sciences Uppsala Sweden

**Keywords:** Dawul Kurundu (*Neolitsea involucrate*), mucilaginous material, natural stabilizer, yoghurt

## Abstract

The incorporation of plant‐derived stabilizers in food processing and preservation has gained considerable industrial interest. The leaf extract of *Neolitsea involucrate*, Dawul Kurundu (DK), has proven to be a potent plant‐derived stabilizing agent in the food industry. However, the potential of utilizing DK leaf extract in the dairy industry has not yet been proven. Thus, the feasibility of incorporating DK leaf extract in set yoghurt production by assessing its physicochemical, sensory, proximate composition, minerals (calcium and phosphorous), and microbial (*Escherichia coli*, yeast, and mold) quality parameters during storage at 4°C up to 21 days was assessed. DK leaf aqueous extracts of 0.4% w/v (T2), 0.6% w/v (T3), and 0.8% w/v (T4) were used for testing with the control sample, 0.6% gelatin (T1). Compared to T1, there were no differences in color, taste, texture, and mouthfeel in all DK leaf extract‐incorporated yoghurts, demonstrating the suitability of using DK leaf extract to replace the gelatin. A decreasing pattern of pH value was observed during 21 days of the storage period in all treatments, whereas total titratable acidity increased significantly with time. Furthermore, the lowest syneresis value was obtained by T4, demonstrating ideal stabilizing properties at higher incorporation levels. The proximate, mineral, and microbial compositions of all treatments showed no significant difference compared to the control. Therefore, overall results revealed that the 0.8% w/v level of DK leaf extract incorporation (T4) could be used as a potent stabilizer in set yoghurt production by allowing the possibility of replacing the gelatin without compromising its organoleptic properties. Improved and efficient methods for extracting the DK leaf extracts by focusing on their potential functional and health effects should be further examined.

## Graphical abstract

In comparison to the control group, no differences in sensory attributes were observed in all yoghurts that incorporated Dawul Kurundu (DK) leaf extract. Over the 21‐day storage period, a decreasing trend in pH values was observed in all treatments, while the total titratable acidity significantly increased over time. The results indicate that incorporating 0.8% w/v of DK leaf extract could serve as an effective stabilizer in the production of set yoghurt, allowing for the potential replacement of gelatin without affecting its sensory properties.

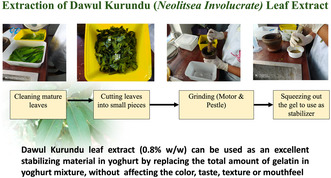

## 1 INTRODUCTION

Recently, the need for more convenient and clean food products with additional benefits has become a rising issue in the food industry. Yoghurt is one of the popular fermented milk‐derived products that has gained substantial attraction among children and adults in Sri Lanka. It is produced from whole, low‐fat, or non‐fat milk. A culture of lactic acid bacteria, including *Lactobacillus delbrueckii* subsp. *bulgaricus* and *Streptococcus thermophilus* is commonly used in yoghurt preparation to convert lactose into lactic acid during fermentation. Stabilizers are also a major ingredient used in yoghurt preparation in order to improve the body and texture of yoghurt, as a rigid gel structure is crucial for optimal customer acceptance.

Gelatin is the most common stabilizer used in yoghurt production. It is regarded as a hydrocolloid with multiple functions and a wide range of applications, including increased protein network density in the microstructure, causing a solid network, providing higher viscosity and less syneresis for the product and improving the textural appearance and mouthfeel of the final product. Gelatin is obtained by partial hydrolysis of collagen derived from animal‐derived raw materials (Emine & Ihsan, 2017; Karim & Bhat, 2008), thus limiting its use for certain consumer niches.

There is a growing interest of people in vegetarianism; thus, the demand for vegetarian foods has increased recently. Due to these factors, the demand for gelatin‐included food products is also declining considerably. Plant‐based polymers are commonly used in the food industry as viscosity enhancers, stabilizers, emulsifiers, binders, and solubilizers. Some plant‐based stabilizers are used in yoghurt production, such as modified gums like xanthan gum and guar gum, carrageenan, alginate, pectin, and modified starch (Emine & Ihsan, 2017; Lal et al., 2006). Further, xanthan and carrageenan gums increase the viscosity and decrease the syneresis of yoghurt samples (Hematyar et al., 2012).

Dawul Kurundu (DK) leaf extracts can be used as plant‐based polymers as they contain mucilaginous material that has the capability of acting as a binding agent in a variety of food products, such as coating foods with mucilaginous material to reduce oil absorption during frying, as a binding material in producing rice noodles, and as a wax coating to extend the postharvest life of lime. However, those attempts are currently not commercialized but proposed for research applications (Kasunmala et al., 2020; Weerasekera & Navaratne, 2015). Nevertheless, mucilaginous material extracted from DK leaves used in traditional culinary dishes for centuries in Sri Lanka proving that it is safe for consumption (Kasunmala et al., 2017). DK leaf extract contains water‐soluble arabinoxylan that can bind free water and increase the water‐holding capacity of the gel network (Jeewanthi & Gunathilake, 2020). Moreover, the main chemical constituent in DK (Arabinoxylan) acts as a probiotic, thus promoting the growth of helpful gut bacteria and increasing gastrointestinal health. And also, it is a form of dietary fiber that fosters the growth of probiotic bacteria, thus improving digestion, enhancing the absorption of food and nutrients, and protecting against hostile bacteria (Weerasekera & Navaratne, 2015). This study aims to evaluate the overall suitability of DK leaf extract as a natural stabilizer to replace gelatin in set yoghurt production concerning its sensory, microbial, proximate, mineral, and physicochemical properties.

## 2 MATERIALS AND METHODS

### 2.1 Experimental design and treatments

The experimental design was a complete randomized design with four treatments and three replicates per treatment. The four treatments are T1, T2, T3, and T4. The inclusion conditions are 0.6% gelatin (T1), and 0.4%, 0.6%, and 0.8% (T2, T3, and T4, respectively) DK leaf extracts.

### 2.2 Extraction of DK leaf extract

Matured DK leaves were selected based on ideal morphological observations and cleaned in the following sequence, tap water, hot water, and then distilled water, to remove any potential impurities. Leaves were cut into smaller pieces and ground by adding 10 mL of distilled water to 30 g of DK leaves using a mortar and pestle in order to extract leaf gel. The extracted gel was squeezed out by using two layers of clean muslin cloth. The collected gel was stored at 4°C in the refrigerator until further use.

### 2.3 Incorporation of DK leaf extract into yoghurts

The skim milk powder (depending on the total solid content of the raw milk, 1%–1.2%) was added to a homogenized raw milk sample to achieve the final total solid content of 12.5% in the raw milk sample. The amount of 10% sugar and 0.6% gelatin (as the control sample) was added to the raw milk sample. The homogenized mixture was pasteurized at 72°C for 15 s using a hot water bath. Then the pasteurized sample was cooled up to 45°C and inoculation was done (following the recommended inoculation rate of 50 U of culture per 500 L of raw milk sample) using a probiotic yoghurt culture (STI12, Chr Hansen, Denmark; composition: *Streptococcus thermophiles*). The amount of 0.007% vanillin was added as the flavoring agent. Thereafter, the sample was filled into food‐grade high‐impact polystyrene cups and incubated at 40–45°C for about 4 h until 4.6 pH. The incubated yoghurt samples were stored in an industrial cool room at a temperature of 4°C for 21 days. The same procedure was employed for the production of yoghurts incorporated with DK leaf extract. The DK leaf extract was added to the homogenized raw milk sample before pasteurization. The percentage of DK leaf extractions into the yoghurts was investigated through a preliminary sensory screening based on appearance, color, texture, taste, mouthfeel, and odor (not presented within this study).

### 2.4 Sensory evaluation of DK leaf extract‐incorporated yoghurts

Sensory evaluation was conducted to select the best level of DK leaf extract that can be incorporated into the yoghurt using thirty‐five untrained panelists after 3 days of refrigerated (4°C) storage. The yoghurts were numbered using random four‐digit numbers and served to the sensory panel at room temperature to evaluate sensory attributes (appearance, color, texture, taste, mouthfeel, and odor). The best treatment was selected using a five‐point hedonic scale test.

### 2.5 Evaluation of the physiochemical properties

The shelf life of the selected treatments was evaluated based on physicochemical parameters such as pH and TTA (total titratable acidity) (AOAC (Association of Office Analytical Chemist), 2005). Syneresis was measured according to the drainage method described by Priyashantha et al. (2019). It was performed using undisturbed yoghurt samples (without mechanical disturbances) on the 6th day of cold storage at 4°C. The syneresis measurement was done using a 25‐mL measuring cylinder by draining out the expelled whey carefully with an average of six samples per treatment (Priyashantha et al., 2019).

### 2.6 Proximate, Mineral & Microbial Analysis of the DK leaf extract incorporated yoghurts

The selected treatments (T1 and T4) were analyzed for fat (Gerber method), total solid, total solid non‐fat, crude protein (Kjeldhal method) according to AOAC (2005), and coliform, yeast, and mold counts (total plate count), calcium, and phosphorous, according to AOAC (2005) on the 6th day of cold storage.

### 2.7 Statistical analysis

Physicochemical, mineral, and microbial analyses were carried out using one‐way analysis of variance (ANOVA) using the Minitab software package (Minitab 19.1) with three replicates. A Tukey's pairwise comparison was used for grouping information. The significance level was established at *p* < .05. The results of the sensory evaluation were analyzed by a Kruskal–Wallis non‐parametric test using the Minitab software package (Minitab 19.1).

## 3 RESULTS AND DISCUSSION

### 3.1 Analysis of sensory evaluation

Sensory evaluation was performed to select the best concentration of DK leaf extract that can be incorporated into the yoghurt based on the most consumer‐preferable treatment. The reactions of the panelists to the quality characters of the product were evaluated during storage at 4°C.

**FIGURE 1:**
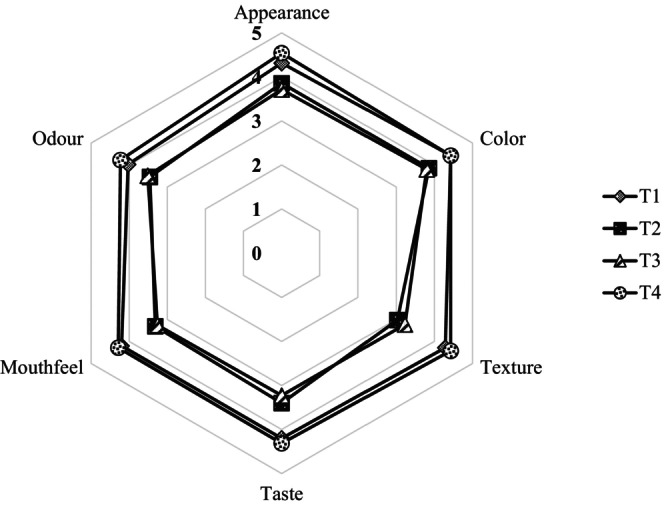
Effect of replacing gelatin with DK leaf extract on the sensory quality of yoghurt.

Appearance, color, texture, taste, mouthfeel, odor, and overall acceptability were analyzed using the Kruskal‐Wallis test. Sensory analysis results (Figure 1) have shown that all four treatments significantly differ from each other (*p* < .05). The T1 and T4 samples had similar mean rank values for appearance, color, taste, and mouthfeel, while the other two treatments had lower mean rank values for all sensory attributes. The highest mean rank for appearance, color, texture, taste, mouthfeel, odor, and overall acceptability was achieved by the (T4) 0.8% DK leaf extract‐incorporated sample (Figure 1).

Jeewanthi and Gunathilake (2020) reported that DK leaves contain water‐soluble arabinoxylan, a hydrocolloid that can provide functional properties to the product, including thickening, gelling, emulsifying, stabilizing, and water‐binding properties, thus modifying the rheology of the food. The reasons for lower sensory values for T2 and T3 when compared to T1 and T4 may be that the concentration of leaf gel is responsible for the syneresis, which is directly related to the water‐holding capacity of the yoghurt. The higher the syneresis, the lower the water‐holding capacity. When the concentration of leaf gel was lower (0.4%; T2) in the yoghurt, it may not have been enough to maintain the required level of syneresis and the water‐holding capacity (Zainoldin & Baba, 2009).

Therefore, as demonstrated in this study, replacing gelatin with DK leaf extract without compromising its sensory properties was feasible, potentially due to its water‐soluble hydrocolloids. Further, the present study could reveal that increasing the level of DK leaf extract results in the enhancement of sensory attributes in yoghurt. It might be the probable reason for the highest value for sensory scores in the T4 sample compared with others.

### 3.2 Analysis of physicochemical parameters

#### 3.2.1 Analysis of pH

**FIGURE 2:**
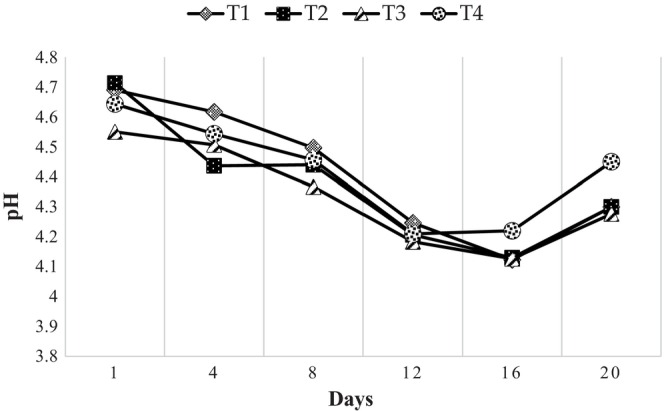
Changes in pH values of yoghurt during the storage period at 4°C.

During the cold storage of yoghurt for 21 days at 4°C, pH values changed significantly. However, the pH values were within the acceptable range for yoghurt (<4.5) during the storage period of all the treatments (SLS 824:1989). According to Lee and Lucey (2010), 4.6 is the isoelectric point of the casein, and the reduction of the net negative charge of the casein leads to a decrease in the electrostatic repulsion between casein molecules, resulting in the formation of a three‐dimensional network consisting of clusters of caseins. This structure provides a solid texture to the gel of the yoghurt, since if the pH is not at an acceptable level, the firm texture of the yoghurt cannot be achieved (Lee & Lucey, 2010). The pH values of all the treatments were reduced until Day 16 during the storage period (Figure 2). The reduction of pH in yoghurt during storage is due to the post‐acidification by lactic acid bacteria by acting upon the remaining lactose content and producing organic acids (Deshwal et al., 2021), which leads to a lowering of pH. During Days 4 and 8, the pH values of all four treatments significantly (*p* < .05) differed (Figure 2). However, there was no significant difference (*p* > .05) in pH values for all four treatments on Days 1, 12, 16, and 20 during the storage period. The increased pH on Day 20 could be seen in all four treatments, and T4 gave the highest mean value. The probable reason for the increase in pH of yoghurt at the end of its shelf life is due to the production of metabolites such as amino acids, bacteriocins, and vitamins, as reported by Mohammadi‐Gouraji et al. (2019).

#### 3.2.2 Analysis of syneresis

**FIGURE 3:**
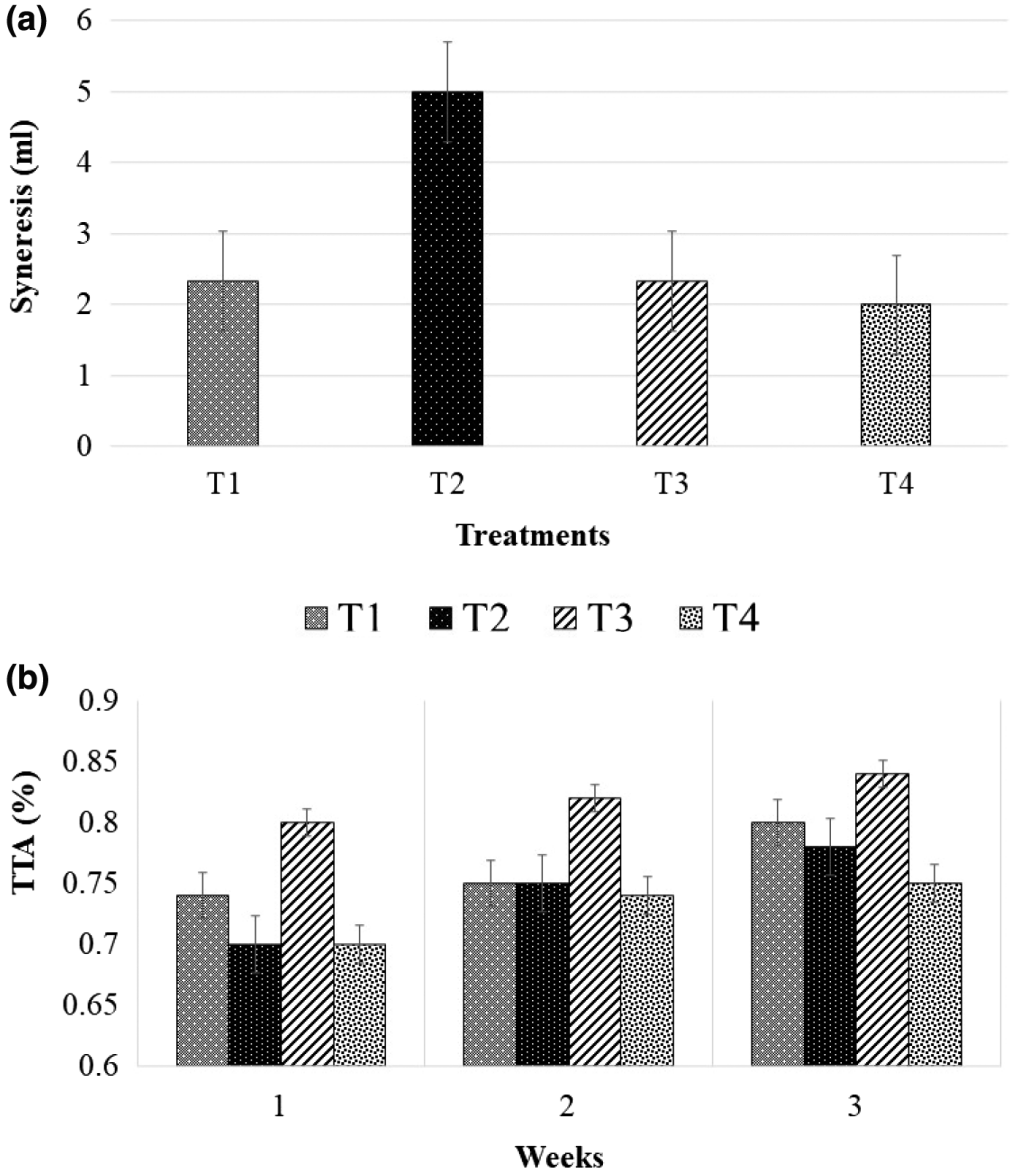
(a) Syneresis values of treatments on the 6th day of the storage period; (b) total titratable acidity of treatments during the three weeks of the storage period.

The syneresis values of all the treatments during the 6th day of the storage period were significantly (*p* < .05) different (Figure 3a). The T2 treatment had the highest syneresis value, whereas the T4 treatment had the lowest syneresis value compared to the control (T1). These results suggest that increasing the level of DK leaf extract is advantageous to reduce syneresis, which will also eventually result in a rigid gel structure in yoghurt due to its hydrocolloid properties. Jeewanthi and Gunathilake (2020) reported that Dawul Kurundu leaves are an advantageous raw material for isolating arabinoxylan, which is a hydrocolloid that improves the properties of the product, including viscosity, texture, emulsion stability, and water‐binding capacity, thus resulting in a rigid gel structure. This might be the probable reason for reducing syneresis by increasing the percentage of DK leaf extract.

Having a lower level of syneresis is preferred by general consumers, and therefore, DK leaf extract‐incorporated yoghurts are likely to have higher consumer demands in terms of lower syneresis since, as reported by many studies, for example, Afoakwa (2014), Joon et al. (2017), and Lee and Lucey (2010), expulsion of whey from the structure can negatively influence consumer acceptance of the product as it can be perceived as a quality defect.

#### 3.2.3 Analysis of total titratable acidity

There was no significant difference in the titratable acidity values of all treatments during the 21‐day storage period (Figure 3b). However, there was a general non‐significant trend of increasing titratable acidity over the course of storage. The T3 treatment had the highest titratable acidity compared to other treatments, whereas T1, T2, and T4 were not significantly different from each other (Figure 3b). The acceptable range for titratable acidity is 0.8%–1.25% (SLS 824:1989) during the storage period in the context of Sri Lanka, and the treatment results as reported in this study were within the acceptable range.

Yousefi and Jafari (2019) discussed that the increase in the TTA of yoghurt during the storage period might be a consequence of the fermentation process by microorganisms and the degradation of lactose into lactic acid. Zainoldin and Baba (2009) reported that pH and titratable acidity are key mechanisms of yoghurt fermentation. With the optimum incubation environment, sugar and protein contents in milk promote the growth of the culture bacteria *Streptococcus thermophilus* rapidly. The reaction of *Streptococcus thermophilus* on milk sugar produces lactic acid, acetaldehyde, diacetyl, and formic acid, resulting in post‐acidification during fermentation (Zainoldin & Baba, 2009). Further, plant‐based hydrocolloids alter acidity. Based on the type of hydrocolloid, the acidity of yoghurt may change; e.g., hydrocolloids that are based on amino acids result in a lower acidity (Yousefi & Jafari, 2019).

### 3.3 Analysis of proximate, mineral, and microbial compositions

According to Table 1, the content of fat, total solids, milk solid non‐fat, crude protein, calcium, and phosphorous in both treatments (control; T1 and selected; T4) was not significantly different. The minimum standard values for fat, milk solid non‐fat, and crude protein for yoghurt are 3.0%, 8.0%, and 2.7%, respectively (SLS 824:1989), confirming the values of T1 and T4 are at an acceptable level (Table 1). The total plate counts of both treatments (T1 vs. T4) were not significantly different (Table 1), illustrating that the microbiological quality of the yoghurts is identical without any potential bactericidal or prebiotic effects. According to the SLS 824 (1989) standard, *Escherichia coli* should be absent in yoghurt, the yeast should not be more than 100 per gram, and mold should not be more than 10 per gram, suggesting that the treatments were in the acceptable range.

**TABLE 1:** Proximate and mineral compositions of yoghurts.

Proximate and mineral compositions of yoghurts		
Composition	Treatment 1 (control)	Treatment 4 (selected)
Fat (%)	4.3	4.2
Total solid (%)	21.2	21.4
Milk solid non‐fat (%)	18.7	17.6
Crude protein (%)	3.2	3.5
Calcium (g/kg)	32.2	40.4
Phosphorous (g/kg)	4.5	4.2

*Escherichia coli*, yeast, mold, and total plate count in yoghurts during storage		
Parameters (CFU/mL)	Treatment 1 (control)	Treatment 4 (selected)
*E. coli*	Not detected	Not detected
Yeast	Not detected	Not detected
Mold	Not detected	Not detected
Total plate counts	3.0 × 10^2^	2.0 × 10^2^

Abbreviation: CFU, Colony forming unit.

## 4 COST COMPARISON

The cost of the major ingredients, including raw milk, sugar, skim milk powder, and vanilla flavor, remains the same for both treatments (T1 and T4). The main factor that influences the changing cost of production is gelatin. DK leaves are a locally available material (wild variety). Hence, there is no defined cost for it at this stage (scale) of production. Therefore, the cost of T4 can be lowered when compared with T1.

## 5 CONCLUSION

The present study demonstrated the feasibility of using DK leaf extract as a stabilizer in the yoghurt industry as a potential replacement for gelatin. It was found that among the tested treatments, 0.8% incorporation (T4) was the best DK leaf extract inclusion level for the yoghurts without compromising the sensory and physicochemical properties. It showed a shelf‐life period of about 21 days without any significant adverse impact on pH and titratable acidity values. The tested quality attributes (proximate and minerals) explain that the incorporation of DK leaves into yoghurt maintains the essential quality attributes in terms of protein, fat, calcium, and phosphorous. Thus, it can be concluded that DK leaf extract could be demonstrated as a potential stabilizer that can be used in the yoghurt industry instead of gelatin. More research work is guaranteed to further investigate the strategies for efficient extraction of the DK leaf or purifying the suitable hydrocolloids to upscale the application of DK leaf extracts in yoghurt production. Moreover, additional studies focusing on nutritional and health benefits are recommended to assess the potential for delivering added health‐promoting benefits to consumers with the incorporation of DK leaf extract as a potent stabilizer.

## AUTHOR CONTRIBUTIONS

**Wimukthika Wijekoon:** Conceptualization (equal); data curation (equal); formal analysis (equal); investigation (equal); methodology (equal); resources (equal); software (equal); validation (equal); visualization (equal); writing – original draft (equal). **Udayagee Kumarasinghe:** Conceptualization (lead); data curation (equal); formal analysis (equal); funding acquisition (equal); investigation (equal); methodology (equal); project administration (lead); resources (equal); software (equal); supervision (equal); validation (equal); writing – review and editing (equal). **Amali Alahakoon:** Conceptualization (equal); methodology (equal); resources (equal); supervision (equal); validation (equal); writing – review and editing (equal). **Shishanthi Jayarathna:** Conceptualization (equal); data curation (equal); formal analysis (equal); methodology (equal); resources (equal); software (equal); supervision (equal); validation (equal); writing – review and editing (equal). **Hasitha Priyashantha:** Conceptualization (lead); formal analysis (equal); funding acquisition (equal); methodology (equal); resources (equal); software (equal); supervision (lead); validation (equal); visualization (equal); writing – review and editing (equal).

## FUNDING INFORMATION

This article received no external funding.

## CONFLICT OF INTEREST STATEMENT

The authors declare no conflict of interest.

## ETHICS STATEMENT

Not applicable.

## Data Availability

The data that support the findings of this study are available from the corresponding author upon reasonable request.
